# Association of Tramadol and Hypoglycemia in Diabetic Asians

**DOI:** 10.3390/jcm7110380

**Published:** 2018-10-24

**Authors:** Shang-Yi Li, Hsin-Hung Chen, Cheng-Li Lin, Su-Yin Yeh, Chia-Hung Kao

**Affiliations:** 1 Department of Otorhinolaryngology, Head and Neck Surgery, Changhua Christian Hospital, Changhua 500, Taiwan; 150614@cch.org.tw; 2 Institute of Medicine and Public Health, Chung Shan Medical University, Taichung 402, Taiwan; 605319@cch.org.tw; 3 College of Nursing and Health Sciences, Dayeh University, Changhua 515, Taiwan; 4 Division of Metabolism Endocrinology, Changhua Christian Hospital, Changhua 500, Taiwan; 5 Division of Metabolism Endocrinology, Nantou Christian Hospital, Nantou 540, Taiwan; 6 Management Office for Health Data, China Medical University Hospital, Taichung 404, Taiwan; orangechengli@gmail.com; 7 College of Medicine, China Medical University, Taichung 404, Taiwan; 8 Department of Healthcare Administration, Asia University, Taichung 413, Taiwan; nch2019@yahoo.com.tw; 9 Graduate Institute of Biomedical Sciences and School of Medicine, College of Medicine, China Medical University, No. 2, Yuh-Der Road, Taichung 404, Taiwan; 10 Department of Nuclear Medicine and PET Center, China Medical University Hospital, Taichung 404, Taiwan; 11 Department of Bioinformatics and Medical Engineering, Asia University, Taichung 404, Taiwan

**Keywords:** tramadol, hypoglycemia, diabetes

## Abstract

To evaluate the association between tramadol and hypoglycemia in diabetic Asians. The data adopted in this study were derived from a subset of the National Health Insurance (NHI) Research Database, which comprises data on one million randomly sampled beneficiaries enrolled in the NHI program. Patients diagnosed with diabetes (according to the International Classification of Diseases, Ninth Revision, Clinical Modification code 250) were identified from claims data between 1998 and 2011. Diabetic patients aged 20 years or older and prescribed tramadol constituted the tramadol group and other diabetic patients without tramadol use constituted the non-tramadol group. For each tramadol case, one non-tramadol control frequency matched according to age (every 5 years), sex and the year of tramadol use was identified. The tramadol group comprised 12,446 patients and non-tramadol group comprised 11,982 patients. During a mean follow-up of 2 years for the patients in the tramadol group and 2.79 years for those in the non-tramadol group, the overall incidences of hypoglycemia (per 1000 person-years) were 7.37 and 3.77, respectively. According to the multivariable analyses, after baseline characteristics were controlled, the tramadol group exhibited a significantly greater risk of hypoglycemia (hazard ratio (HR) = 1.34, 95% confidence interval (CI) = 1.05–1.71) compared with the non-tramadol group. Tramadol use increases hypoglycemia in diabetic Asians. Greater attention must be paid to diabetic Asians with tramadol use.

## 1. Introduction

Hypoglycemia poses a challenge in managing diabetes. The action to control cardiovascular risk in diabetes study showed that hypoglycemia caused considerable mortalities [1]. In addition, the action in diabetes and vascular disease: Preterax and diamicron modified release controlled evaluation indicated that hypoglycemia increases microvascular as well as macrovascular comorbidities and mortalities [2]. Clinical symptomatic hypoglycemia causes cardiovascular events, all-cause hospitalization and mortality [3]. The risk of dementia increased in older diabetic patients [4]. Cognitive impairment was significantly associated with severe hypoglycemia [5]. In 2013, the American Diabetes Association and the Endocrine Society published a consensus report called “Hypoglycemia and Diabetes [6].” Thus, how to avoid hypoglycemic attacks in diabetic patients has been extensively discussed worldwide.

According to Real-life Effectiveness and Care Patterns of Diabetes Management [7], 29.4% of diabetic patients suffered a hypoglycemic attack. Tramadol is one of the medications prescribed by the American Diabetes Association for treating painful diabetic peripheral neuropathy [8]. However, tramadol therapy with non-cancer pain could increase the risk of hypoglycemia and lead to hospitalization [9]. A previous study indicated that hypoglycemia is one of the major reasons for emergency hospitalizations for adverse drug events in older Americans [10], reporting that insulin, instead of tramadol, caused emergency hospitalizations in the United States of America. The objective of the present study was to evaluate the association between tramadol and hypoglycemia in diabetic Asians.

## 2. Methods

### 2.1. Data Source

The National Health Insurance (NHI) program in Taiwan was implemented in 1995 and provides comprehensive medical care, including ambulatory and inpatient care, to nearly 100% of Taiwan’s population, which is approximately 23.72 million people [11]. The National Health Insurance Research Database (NHIRD) contains historical reimbursement claims data from the NHI program and is maintained and managed by the National Health Research Institute. The data used in this study were derived from a subset of the NHIRD, which comprises data on one million randomly sampled beneficiaries enrolled in the NHI program from 1996 to 2000. All records of these individuals from 1996 to 2011 were collected. The data files were de-identified and scrambled before being released to researchers. Therefore, this database cannot be used to query the data alone to identify individuals at any level. The diagnoses and procedures were coded according to the International Classification of Diseases, Ninth Revision, Clinical Modification (ICD-9-CM).

### 2.2. Sampled Participants

Patients diagnosed with diabetes (ICD-9-CM code 250) were identified from the claims data between 1998 and 2011. Diabetic patients aged 20 years or older and prescribed tramadol constituted the tramadol group and those who did not use tramadol constituted the non-tramadol group. The initial tramadol treatment date was defined as the index date. For each tramadol case, one non-tramadol control frequency matched according to age (every 5 years), sex and the year of tramadol use was identified. Controls were randomly assigned the same index date as the tramadol group. Patients in both groups with a history of hypoglycemia (ICD-9-CM codes 251.0, 251.1 and 251.2) or with incomplete information were excluded.

### 2.3. Outcome

Both the tramadol and non-tramadol groups were followed from the index date to the date of hypoglycemia diagnosis, withdrawal from the NHI program, censoring because of death, or end date of the database (31 December 2011).

### 2.4. Comorbidities and Medications 

The records of comorbidities and medications were obtained before the index date. Comorbidities included acute myocardial infarction (ICD-9-CM code 410), chronic kidney disease (ICD-9-CM codes 580–589), stroke (ICD-9-CM codes 430–438), hypertension (ICD-9-CM codes 401–405), cancer (ICD-9-CM codes 140–208) and alcohol-related illness (ICD-9-CM codes 291, 303, 305, 571.0, 571.1, 571.2, 571.3, 790.3, A215 and V11.3). Medications included antidiabetic drugs (including alpha-glucosidase inhibitors, sulfonylurea, metformin, thiazolidinedione, insulin and dipeptidyl peptidase 4), statins, aspirin, nonsteroidal anti-inflammatory drugs, antihypertensive agents (including angiotensin-converting enzyme inhibitors, angiotensin II receptor blockers (AIIRBs), calcium channel blockers (dihydropyridine) (CCB (DHP)), α-blockers, β-blockers, CCB (non-DHP), loop diuretics and thiazides), antiarrhythmics, serotonin reuptake inhibitors, benzodiazepine, tricyclic antidepressants (TCA), serotonin-norepinephrine reuptake inhibitors (SNRI) and other opioid.

### 2.5. Statistical Analysis

The distributions of age, sex, comorbidities and medications between the groups with and without tramadol use were compared using chi-square tests for categorical variables and *t* tests for continuous variables. The incidence densities (per 1000 person-years) of hypoglycemia were estimated in both the tramadol and non-tramadol groups. Univariable and multivariable Cox proportional hazards regression models were employed to estimate hazard ratios (HRs) and 95% confidence intervals (CIs) for hypoglycemia in in patients in the tramadol group compared with those in the non-tramadol group. Baseline characteristic variables such as sex, age, comorbidities and medications were included in the multivariate model for adjustment. In the multivariable model, antidiabetic drugs, including sulfonylurea, metformin and insulin and antihypertensive agents, including loop diuretics, attained significance. Additional data analysis was performed to evaluate the interaction among tramadol use, sulfonylurea, metformin, insulin and loop diuretics. The cumulative incidences of hypoglycemia between the tramadol and non-tramadol groups were assessed using the Kaplan–Meier method and the differences were assessed using a log-rank test. All statistical analyses were performed using the SAS statistical package (Version 9.3 for Windows; SAS Institute, Inc., Cary, NC, USA). Statistical significance was accepted at *p* < 0.05.

### 2.6. Data Availability Statement

All data and related metadata were deposited in an appropriate public repository. The data on the study population that were obtained from the NHIRD (http://w3.nhri.org.tw/nhird//date_01.html) are maintained in the NHIRD (http://nhird.nhri.org.tw/). The National Health Research Institutes (NHRI) is a nonprofit foundation established by the government.

### 2.7. Ethics Statement

The NHIRD encrypts patient personal information to protect privacy and provides researchers with anonymous identification numbers associated with relevant claims information, including sex, date of birth, medical services received and prescriptions. Patient consent is not required to access the NHIRD. This study was approved by the Institutional Review Board (IRB) of China Medical University (CMUH104-REC2-115-CR3). The IRB specifically waived the consent requirement.

## 3. Results

In this study, the tramadol group comprised 12,446 patients and non-tramadol group comprised 11,982 patients (Table 1). Among the patients in the tramadol group, 56.9% were older than 65 years and 50.8% were women. The mean ages of the patients in the tramadol and non-tramadol groups were 66.3 (standard deviation (SD) = 12.6) and 65.4 (SD = 12.2) years, respectively. Diabetic patients in the tramadol group were more likely to have acute myocardial infarction, chronic kidney disease, stroke, hypertension, cancer or alcohol-related diseases (*p* < 0.001) compared with those in the non-tramadol group. At the baseline, all the medications were more prevalent in the tramadol group (*p* < 0.001) compared with the non-tramadol group.

**Figure 1:**
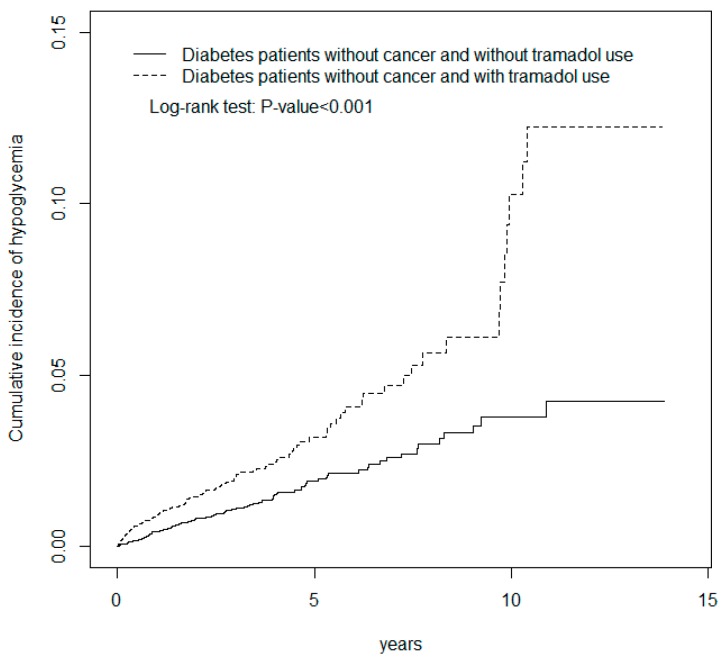
Cumulative incidence of hypoglycemia for patients with (dashed line) or without (solid line) tramadol use among diabetes patients without cancer.

During the mean follow up of 2 years for the tramadol group and 2.79 years for the non-tramadol group, the overall incidences of hypoglycemia (per 1000 person-years) were 7.37 and 3.77, respectively (Table 2). According to the multivariable analyses, after baseline characteristics were controlled, the patients in the tramadol group exhibited a significantly greater risk of hypoglycemia (HR = 1.32, 95% CI = 1.03–1.68) compared with those in the non-tramadol group. The incidence of hypoglycemia increased with age in both groups. The relative risk of hypoglycemia in the sex-specific tramadol group to non-tramadol group was significant for men (HR = 1.66, 95% CI = 1.10–2.50). We analyzed the association between tramadol use and the risk of hypoglycemia stratified according to cancer and observed an approximate 1.33-fold hypoglycemia risk in patients with tramadol use and without cancer (HR = 1.33, 95% CI = 1.03–1.71). The cumulative incidence of hypoglycemia (log-rank *p* < 0.001) was significantly higher for patients with tramadol use and without cancer than it was for patients without tramadol use and without cancer (Figure 1). The sex-specific hazard of hypoglycemia in patients with tramadol use and without cancer relative to that in patients without tramadol use and without cancer was significant for men (HR = 1.60, 95% CI = 1.05–2.43). 

Table 3 shows the results of the univariable and multivariable Cox proportional hazard analyses conducted for evaluating the association between hypoglycemia and tramadol use. The adjusted HR of hypoglycemia development was a 1.47-fold increase for men compared with that of women (HR = 1.36, 95% CI = 1.05–1.76) and a 1.05-fold increase (95% CI = 1.04–1.06) with age (every 1 year). The risk of hypoglycemia was higher for patients with chronic kidney disease (HR = 1.36, 95% CI = 1.06–1.75) and medications of sulfonylurea (HR = 1.72, 95% CI = 1.18–2.51), metformin (HR = 1.74, 95% CI =1.20–2.53), insulin (HR = 1.86, 95% CI = 1.32–2.61) and loop diuretics (HR = 1.32, 95% CI = 1.01–1.73). Table 4 shows the joint effect of tramadol, sulfonylurea, metformin, insulin, loop diuretics, benzodiazepine, TCA, SNRI and other opioid use on the risk of hypoglycemia. Compared with the patients without tramadol and sulfonylurea use, the patients with tramadol and sulfonylurea use exhibited an adjusted HR of 2.64 (95% CI = 1.56–4.44). The HR increased to 2.24 (95% CI = 1.49–3.36) for the patients with tramadol and insulin use. The risk of hypoglycemia for patients in the tramadol group was increased with metformin (HR = 2.23, 95% CI = 1.39–3.59) and loop diuretics use (HR = 1.71, 95% CI = 1.21–2.43). Compared with the patients without tramadol and TCA use, the patients with tramadol and TCA use exhibited an adjusted HR of 1.61 (95% CI = 1.05–2.44).

## 4. Discussion

### 4.1. Tramadol and Hypoglycemia in Diabetes

Tramadol is one of the medications prescribed by the American Diabetes Association for treating painful diabetic peripheral neuropathy [8]. Table 1 shows that among the patients in the tramadol group, 56.9% were older than 65 years. The mean ages of the patients in the tramadol and non-tramadol groups were 66.3 (SD = 12.6) and 65.4 (SD = 12.2) years, respectively. The diabetic patients in the tramadol group were more likely to have acute myocardial infarction, chronic kidney disease, stroke, hypertension, or cancer (*p* < 0.001) compared with those in the non-tramadol group. At the baseline, all the medications were more prevalent in the tramadol group (*p* < 0.001) compared with the non-tramadol group. This finding is consistent with that of previous studies [3,9,10,12]. Table 2 shows that the overall incidences of hypoglycemia (per 1000 person-years) in the tramadol and non-tramadol groups were 7.37 and 3.77, respectively. The results of the multivariable analyses indicated that the patients in the tramadol group exhibited a significantly higher risk of hypoglycemia (HR = 1.34, 95% CI = 1.05–1.71) compared with those in the non-tramadol group. Some possible mechanisms could explain that tramadol induced hypoglycemia. A previous study conducted on mice and rats indicated that serotonin increased insulin concentration and induced β-endorphin release to stimulate muscle glucose utilization. The pathway activates μ-opioid receptors [13,14]. Another study indicated that μ-opioid receptor activation reduced plasma glucose concentrations through an insulin-independent mechanism [15]. These possible mechanisms explained our finding (Table 2 and Figure 1), which indicates an approximate 1.33-fold hypoglycemia risk in the patients with tramadol use and without cancer (HR = 1.33, 95% CI = 1.03–1.71) and a significantly higher cumulative incidence of hypoglycemia (log-rank *p* < 0.001) in the patients with tramadol use and without cancer.

### 4.2. Tramadol Combined with Other Medications in Diabetes

As shown in Table 3, the risk of hypoglycemia was higher for the diabetic patients with the medications of sulfonylurea (HR = 1.76, 95% CI = 1.19–2.60), metformin (HR = 1.66, 95% CI = 1.14–2.43), insulin (HR = 1.78, 95% CI = 1.26–2.52) and loop diuretics (HR = 1.33, 95% CI = 1.01–1.76) but lower for those with only AIIRBs (HR = 0.71, 95% CI = 0.54–0.93). Regarding diabetes treatment, sulfonylurea and insulin were reported to cause hypoglycemia, which may explain the increased risk of hypoglycemia in the patients evaluated in the current study. However, as shown in Table 4, combining tramadol with metformin resulted in increased hypoglycemia (HR = 2.26, 95% CI = 1.40–3.62). Guidelines for diabetes treatment from different countries frequently state that metformin does not increase hypoglycemia. Metformin activates adenosine monophosphate-activated protein kinase in the liver and muscles to improve glucose and lipid metabolism in diabetic humans; thus, metformin increases hepatic insulin sensitivity [16]. Another study revealed that tramadol enhances hepatic insulin sensitivity [17]. One possible explanation is that combining tramadol with metformin increased hepatic insulin sensitivity, resulting in hypoglycemia in diabetes. We suggest reducing the dose of metformin to avoid a hypoglycemic attack if the combination therapy with tramadol is necessary in diabetic Asian. Regarding the loop diuretics conducted in this study to increase hypoglycemia, only one table shows established and putative drugs that cause hypoglycemia from Williams Textbook of Endocrinology [18]. Diuretics was one of the putative drugs in this textbook. The Captopril Prevention Project suggested that conventional treatment with β-blocker and diuretics resulted in new-onset diabetes [19], which is an indirect effect for explaining our finding. Another finding in the current study is that tramadol plus AIIRBs resulted in less hypoglycemia. Several studies conducted on AIIRB have shown that these types of medication, including valsartan, losartan and candesartan, reduce new-onset diabetes. One study indicated the possible mechanism in which AIIRBs attenuate renin-angiotensin system activities induced by hyperglycemia or dyslipidemia. The net result in the islet cells was to preserve islet cells and avoid hyperglycemia instead of hypoglycemia [20].

Tramadol is prescribed as the active synthetic opioid analgesic drug in central organ and it can cause hypoglycemia after a blunted autonomic counter-regulatory reaction to diabetic patients. Tramadol can lower plasma glucose in diabetic rats because of an activation of mu opioid receptors (MOR) and this kind of activation results in a reduction of hepatic gluconeogenesis and also an increase of glucose utilization in peripheral tissue [21]. In the other study, tramadol has the effects on the central nervous system to interfere hepatic glucose utilization and it may cause the possibility of hypoglycemia [17]. Tramadol related to hypoglycemia may be based on the diabetic type such as beta cell failure or insulin resistance or the diabetic hormonal regulatory mechanism [22].

## 5. Strengths and Limitations

The strengths of our study are its population-based design, generalizability of findings and use of population-based data and the National Health Insurance Research Database (NHIRD) records with a very large sample size including study and control cohorts. In addition, NHIRD covers a highly representative sample of Taiwan’s general population because the reimbursement policy is universal and operated by a single-buyer, the government in Taiwan. The accuracy of medical coding in the claims data may affect the data validity. All insurance claims should be scrutinized by medical reimbursement specialists and peer review. If these doctors or hospitals make wrong diagnoses or coding, they will be punished with a lot of penalties. Therefore, the diagnoses or coding in this study were highly reliable.

There are some study limitations. First, we could not collect other factors associated with hypoglycemic risk, such as ethanol use, or other lifestyle factors, such as diet and exercise pattern, from the NHIRD. Second, we could not record herbal or other traditional diabetes treatments, which may induce a risk of hypoglycemic attack. Third, bitter melon and okra are widely used throughout Asia as a staple food but it was also a limitation for our data form NHIRD. Fourth, poor compliance or adherence for medication use could be a factor for hypoglycemia but it was also difficult to use the National Health Insurance Research Database (HNIRD) data for analysis.

## 6. Conclusions

Tramadol use increases hypoglycemic risk in diabetic patients. In this study, we should pay more attention to prevent hypoglycemia attack in those high risk diabetic patients who were older, with chronic kidney disease and prescribed medications such as insulin, sulfonylurea, metformin and loop diuretics in Asia.

## Figures and Tables

**Table 1:** Comparison of baseline characteristics between diabetes patients with and without tramadol use.

Variables	Tramadol				*p* Value
	No		Yes		
	*n* = 11,982		*n* = 12,446		
	*n*	(%)	*n*	(%)	
Age, year					0.05
≤34	1311	10.9	1311	10.5	
35–64	4058	33.9	4058	32.6	
65+	6613	55.2	7077	56.9	
Mean (SD)	65.4	12.2	66.3	12.6	<0.001 ^†^
Sex					0.99
Female	6085	50.8	6322	50.8	
Male	5897	49.2	6124	49.2	
Comorbidity					
Acute myocardial infarction	227	1.89	354	2.84	<0.001
Chronic kidney disease	1931	16.1	3380	27.2	<0.001
Stroke	1712	14.3	2120	17.0	<0.001
Hypertension	9343	78.0	9932	79.8	<0.001
Cancer	618	5.16	2120	17.0	<0.001
Alcohol-related diseases	629	5.25	1267	10.2	<0.001
Medication					
Alpha-glucosidase inhibitors	1899	15.9	3360	27.0	<0.001
Sulfonylurea	7329	61.2	9241	74.3	<0.001
Metformin	7935	66.2	9571	76.9	<0.001
Thiazolidinediones	1534	12.8	2998	24.1	<0.001
Others	83	0.69	227	1.82	<0.001
Insulin	6416	53.6	9244	74.3	<0.001
Dpp4	411	3.43	819	6.58	<0.001
Statin	5272	44.0	6259	50.3	<0.001
Aspirin	7102	59.3	8379	67.3	<0.001
NSAID	11,574	96.6	12,316	99.0	<0.001
ACEI	7086	59.1	8278	66.5	<0.001
AIIRB	5301	44.2	6516	52.4	<0.001
CCB (DHP)	8129	67.8	9316	74.9	<0.001
α-Blockers	3490	29.1	4330	34.8	<0.001
β-Blockers	7834	65.4	9096	73.1	<0.001
CCB (non-DHP)	3927	32.8	5055	40.6	<0.001
Loop diuretics	4769	39.8	7695	61.8	<0.001
Thiazides	6914	57.7	7986	64.2	<0.001
Antiarrhythmics	461	3.85	688	5.53	<0.001
Serotonin reuptake inhibitors	334	2.79	556	4.47	<0.001
Benzodiazepine	10,308	86.0	11,768	94.6	<0.001
TCA	617	5.15	1285	10.3	<0.001
SNRI	133	1.11	330	2.65	<0.001
Other opioid	8627	72.0	10,916	87.7	<0.001
Year					
1998			1555	12.5	
1999			2197	17.7	
2000			1886	15.2	
2001			977	7.85	
2002			864	6.94	
2003			734	5.90	
2004			845	6.79	
2005			710	5.70	
2006			616	4.95	
2007			618	4.97	
2008			532	4.27	
2009			319	2.56	
2010			118	0.95	

Chi-square test; ^†^ *t*-test; AIIRB, angiotensin II receptor blocker; CCB (DHP), calcium channel blockers (dihydropyridine); SD, standard deviation; TCA, tricyclic antidepressants; SNRI, serotonin-norepinephrine reuptake inhibitors; Dpp4, dipeptidyl peptidase-4 inhibitors; NSAID, non-steroidal anti-inflammatory drug; ACEI, angiotension-converting enzyme inhibitors.

**Table 2:** Comparisons of hypoglycemia incidences between diabetes patients with and without tramadol use and associated hazard ratios by age, sex and comorbidity.

Variables	Tramadol						Crude HR (95% CI)	Adjusted HR ^†^ (95% CI)
	No			Yes				
	Event	PY	Rate ^#^	Event	PY	Rate ^#^		
All	126	33,465	3.77	183	24,838	7.37	1.94 (1.55, 2.44) ***	1.32 (1.03, 1.68) *
Age, years								
≤49	2	4021	0.05	11	3162	3.48	7.70 (1.70, 34.9) **	4.10 (0.70, 24.2)
50–64	20	11,696	1.71	38	8432	4.51	2.66 (1.54, 4.60) ***	1.62 (0.90, 2.92)
65+	104	17,748	5.86	134	13,244	10.1	1.71 (1.32, 2.21) ***	1.26 (0.95, 1.66)
Sex								
Female	83	16,658	4.98	107	13,292	8.05	1.61 (1.21, 2.15) **	1.17 (0.86, 1.60)
Male	43	16,807	2.56	76	11,546	6.58	2.56 (1.75, 3.74) ***	1.66 (1.10, 2.50) *
With cancer								
All	4	1324	3.02	18	2905	6.19	1.88 (0.63, 5.57)	1.53 (0.49, 4.78)
Age, years								
≤49	0	43	0.00	1	304	3.29	-	-
50-64	0	378	0.00	8	1014	7.89	-	-
65+	4	904	4.43	9	1588	5.67	1.24 (0.38, 4.07)	1.26 (0.33, 4.79)
Sex								
Female	1	744	4.03	10	1352	7.39	1.72 (0.47, 6.30)	2.05 (0.44, 9.54)
Male	3	581	1.72	8	1553	5.15	2.60 (0.32, 20.9)	1.92 (0.18, 20.2)
Without cancer								
All	122	32,140	3.80	165	21,932	7.52	1.97 (1.56, 2.50) ***	1.33 (1.03, 1.71) *
Age, years								
≤49	2	3978	0.50	10	2858	3.50	7.89 (1.72, 36.2) **	4.61 (0.75, 28.3)
50–64	100	11,318	1.77	30	7417	4.04	2.31 (1.30, 4.09) **	1.39 (0.75, 2.58)
65+	10	16,844	5.94	125	11,656	10.7	1.79 (1.37, 2.34) ***	1.30 (0.98, 1.73)
Sex								
Female	80	15,914	5.03	97	11,939	8.12	1.62 (1.20, 2.18) **	1.20 (0.87, 1.65)
Male	42	16,226	2.59	68	9992	6.80	2.60 (1.77, 3.84) ***	1.60 (1.05, 2.43) *

Rate ^#^, incidence rate, per 1000 person-years; Crude HR, relative hazard ratio; Adjusted HR ^†^, multivariable analysis including age, sex, comorbidities of acute myocardial infarction, chronic kidney disease, stroke, hypertension and medications of alpha-glucosidase inhibitors, sulfonylurea, metformin, thiazolidinediones, insulin, others, aspirin, ACEI, angiotensin II receptor blocker (AIIRB), calcium channel blockers (dihydropyridine) (CCB (DHP)), α-Blockers, β-Blockers, CCB (non-DHP), loop diuretics, thiazides, antiarrhythmics, serotonin reuptake inhibitors, benzodiazepine and TCA; * *p* < 0.05, ** *p* < 0.01, *** *p* < 0.001; CI, confidence interval; HR, hazard ratio; PY, person-years; TCA, tricyclic antidepressants; ACEI, angiotension-converting enzyme inhibitors.

**Table 3:** Cox model with hazard ratios and 95% confidence intervals of hypoglycemia associated with tramadol use and covariates among diabetes patients without cancer.

Variable	Crude		Adjusted ^†^	
	HR	(95% CI)	HR	(95% CI)
Gender (women vs. men)	1.52	(1.19, 1.92) ***	1.36	(1.05, 1.76) *
Age, years	1.05	(1.04, 1.06) ***	1.05	(1.04, 1.06) *
Baseline comorbidities (yes vs. no)				
Acute myocardial infarction	1.92	(1.08, 3.43) *	1.18	(0.66, 2.11)
Chronic kidney disease	1.99	(1.56, 2.54) ***	1.36	(1.06, 1.75) *
Stroke	1.67	(1.25, 2.22) ***		
Hypertension	2.07	(1.46, 2.95) ***	0.83	(0.53, 1.30)
Cancer	0.94	(0.61, 1.46)	0.75	(0.48, 1.16)
Alcohol-related diseases	0.93	(0.58, 1.49)		
Medication (yes vs. no)				
Tramadol	1.97	(1.56, 2.50) ***	1.32	(1.03, 1.68) *
Alpha-glucosidase inhibitors	2.01	(1.55, 2.60) ***	1.20	(0.90, 1.58)
Sulfonylurea	3.09	(2.19, 4.34) ***	1.72	(1.18, 2.51) **
Metformin	2.71	(1.95, 3.77) ***	1.74	(1.20, 2.53) **
Thiazolidinediones	1.64	(1.24, 2.16) ***	0.97	(0.73, 1.31)
Others	2.41	(1.19, 4.86) *	1.53	(0.78, 3.01)
Insulin	3.28	(2.42, 4.45) ***	1.86	(1.32, 2.61) **
Dpp4	0.94	(0.42, 2.13)		
Statin	1.07	(0.85, 1.36)		
Aspirin	1.46	(1.14, 1.87) **	0.82	(0.62, 1.08)
NSAID	1.83	(0.81, 4.14)		
ACEI	2.18	(1.66, 2.87) ***	1.23	(0.89, 1.70)
AIIRB	1.32	(1.04, 1.68) *	0.72	(0.55, 0.94) *
CCB (DHP)	2.18	(1.62, 2.94) ***	1.38	(0.96, 1.99)
α-Blockers	1.69	(1.34, 2.14) ***	1.24	(0.95, 1.62)
β-Blockers	1.64	(1.25, 2.14) ***	1.05	(0.78, 1.42)
CCB (non-DHP)	1.64	(1.30, 2.07) ***	1.05	(0.82, 1.35)
Loop diuretics	2.38	(1.86, 3.03) ***	1.32	(1.01, 1.73) *
Thiazides	1.85	(1.43, 2.39) ***	1.03	(0.77, 1.38)
Antiarrhythmics	1.87	(1.19, 2.95) **	1.22	(0.77, 1.95)
Serotonin reuptake inhibitors	1.64	(1.00, 2.67) *	1.33	(0.81, 2.18)
Benzodiazepine	1.92	(1.23, 2.99) **	0.97	(0.60, 1.55)
TCA	1.47	(1.03, 2.11) *	1.05	(0.73, 1.52)
SNRI	1.42	(0.67, 3.00)		
Other opioid	1.26	(0.95, 1.66)		

Crude HR, relative hazard ratio; Adjusted HR ^†^, multivariable analysis including age, sex and comorbidities of acute myocardial infarction, chronic kidney disease, stroke, hypertension and medications of alpha-glucosidase inhibitors, sulfonylurea, metformin, thiazolidinediones, insulin, others, aspirin, ACEI, AIIRB, calcium channel blockers (dihydropyridine) (CCB (DHP)), α-Blockers, β-Blockers, CCB (non-DHP), loop diuretics, thiazides, antiarrhythmics, serotonin reuptake inhibitors, benzodiazepine and TCA; * *p* < 0.05, ** *p* < 0.01, *** *p* < 0.001; CI, confidence interval; HR, hazard ratio; AIIRB, angiotensin II receptor blockers; TCA, tricyclic antidepressants; SNRI, serotonin-norepinephrine reuptake inhibitors; Dpp4, dipeptidyl peptidase-4 inhibitors; NSAID, non-steroidal anti-inflammatory drug; ACEI, angiotension-converting enzyme inhibitors.

**Table 4:** Cox proportional hazard regression analysis for the risk of hypoglycemia-associated tramadol use with joint effect of medication.

Variables		Event	PY	Rate ^&^	Adjusted HR ^†^ (95% CI)	*p*-Value ^#^
Tramadol use	Sulfonylurea					0.55
No	No	19	12,188	1.56	1 (Reference)	
No	Yes	107	21,276	5.03	2.11 (1.26, 3.54) *	
Yes	No	21	6315	3.33	1.91 (1.02, 3.57) ***	
Yes	Yes	162	18,522	8.75	2.64 (1.56, 4.44) *	
Tramadol use	Metformin					0.29
No	No	25	11,954	2.09	1 (Reference)	
No	Yes	101	21,510	4.70	1.65 (1.03, 2.64) *	
Yes	No	17	6169	2.76	1.15 (0.62, 2.15)	
Yes	Yes	166	18,669	8.89	2.23 (1.39, 3.59) ***	
Tramadol use	Insulin					0.07
No	No	36	16,590	2.17	1 (Reference)	
No	Yes	90	16,876	5.33	1.57 (1.05, 2.37) *	
Yes	No	15	7087	2.12	0.91 (0.50, 1.68)	
Yes	Yes	168	17,751	9.46	2.24 (1.49, 3.36) ***	
Tramadol use	Loop diuretics					0.78
No	No	62	21,994	2.82	1 (Reference)	
No	Yes	64	11,471	5.58	1.24 (0.86, 1.81)	
Yes	No	46	10,451	4.40	1.24 (0.83, 1.83)	
Yes	Yes	137	14,386	9.52	1.71 (1.21, 2.43) **	
Tramadol use	Benzodiazepine					0.39
No	No	16	5659	2.83	1 (Reference)	
No	Yes	110	27,806	3.96	0.89 (0.52, 1.55)	
Yes	No	5	1457	3.43	1.00 (0.37, 2.76)	
Yes	Yes	178	23,381	7.61	1.22 (0.70, 2.13)	
Tramadol use	TCA					0.06
No	No	123	31,876	3.86	1 (Reference)	
No	Yes	3	1589	1.89	0.37 (0.12, 1.18)	
Yes	No	153	22,032	6.94	1.24 (0.96, 1.60)	
Yes	Yes	30	2806	10.7	1.61 (1.05, 2.44) *	
Tramadol use	SNRI					0.43
No	No	124	33,196	3.74	1 (Reference)	
No	Yes	2	270	7.42	1.73 (0.42, 7.10)	
Yes	No	178	24,166	7.37	1.3 6 (1.06, 1.74) *	
Yes	Yes	5	672	7.44	1.24 (0.49, 3.10)	
Tramadol use	Other opioid					0.05
No	No	42	10,935	3.84	1 (Reference)	
No	Yes	84	22,530	3.73	0.82 (0.56, 1.21)	
Yes	No	21	3240	6.48	1.19 (0.70, 2.03)	
Yes	Yes	162	21,598	7.50	1.16 (0.79, 1.69)	
Tramadol use	NSAID					0.25
No	No	4	1808	2.21	1 (Reference)	
No	Yes	122	31,657	3.85	1.43 (0.52, 3.94)	
Yes	No	3	287	10.4	4.39 (0.98, 19.8)	
Yes	Yes	180	24,551	7.33	1.88 (0.68, 5.20)	

Rate ^&^, incidence rate, per 1000 person-years; ^†^ Model was mutually adjusted for age, sex, comorbidities of acute myocardial infarction, chronic kidney disease, stroke, hypertension and medications of alpha-glucosidase inhibitors, sulfonylurea, metformin, thiazolidinediones, insulin, others, aspirin, ACEI, AIIRB, calcium channel blockers (dihydropyridine) (CCB (DHP)), α-Blockers, β-Blockers, CCB (non-DHP), loop diuretics, thiazides, antiarrhythmics, serotonin reuptake inhibitors, benzodiazepine and TCA. ^#^ *p*-value for interaction, * *p* < 0.05, ** *p* < 0.01, *** *p* < 0.001; CI, confidence interval; HR, hazard ratio; AIIRB, angiotensin II receptor blockers; PY, person-years; TCA, tricyclic antidepressants; NSAID, non-steroidal anti-inflammatory drug; ACEI, angiotension-converting enzyme inhibitors; SNRI, serotonin-norepinephrine reuptake inhibitors.
